# O^6^-methylguanine-DNA methyltransferase as a prognostic and predictive marker for basal-like breast cancer treated with cyclophosphamide-based chemotherapy

**DOI:** 10.3892/ol.2014.1985

**Published:** 2014-03-20

**Authors:** SAYURI ISONO, MAKOTO FUJISHIMA, TATSUYA AZUMI, YUKIHIKO HASHIMOTO, YOSHIFUMI KOMOIKE, MASAO YUKAWA, MASAHIRO WATATANI

**Affiliations:** 1 Department of Surgery, Faculty of Medicine, Kinki University, Osaka-Sayama, Osaka 589-8511, Japan; 2 Department of Gastroenterological and Breast Surgery, Osaka Prefectural Medical Center for Respiratory and Allergic Diseases, Habikino, Osaka 583-8588, Japan; 3 Department of Breast and Endocrine Surgery, Faculty of Medicine Nara Hospital, Kinki University, Ikoma, Nara 630-0293, Japan

**Keywords:** O^6^-methylguanine-DNA methyltransferase, basal-like breast cancer, cyclophosphamide, predictive marker

## Abstract

The O^6^-methylguanine-DNA methyltransferase (MGMT) protein protects cells from alkylating agents by removing alkyl groups from the O^6^-position of guanine. However, its effect on DNA damage induced by cyclophosphamide (CPM) is unclear. The present study investigated whether MGMT expression was correlated with prognosis in patients with breast cancer that was managed according to a common therapeutic protocol or treated with CPM-based chemotherapy. The intrinsic subtypes and MGMT protein expression levels were assessed in 635 consecutive patients with breast cancer using immunohistochemistry. In total, 425 (67%) luminal A, 95 (15%) luminal B, 47 (7%) human epidermal growth factor receptor-2^+^/estrogen receptor^−^ (HER2^+^/ER^−^) and 48 (8%) basal-like subtypes were identified. Of these, MGMT positivity was identified in 398 (63%) of 635 breast cancers; 68% of luminal A, 67% of luminal B, 30% of HER2^+^/ER^−^ and 46% of basal-like subtypes were positive. The overall survival (OS) and disease-free survival (DFS) rates did not significantly differ according to the MGMT status among patients with luminal A, luminal B or HER2^+^/ER^−^ subtypes, and patients with MGMT-negative basal-like cancers tended to have a longer DFS, but not a significantly longer OS time. CPM-containing chemotherapy was administered to 26%, 40%, 47% and 31% of patients with luminal A, luminal B, HER2^+^/ER^−^ and basal-like tumors, respectively. Although the MGMT status and clinical outcomes of patients with the luminal A, luminal B or HER2^+^/ER^−^ subtypes treated with CPM were not significantly correlated, the patients with MGMT-negative basal-like tumors who received CPM exhibited significantly improved DFS and OS compared with the CPM-treated patients with MGMT-positive tumors. MGMT may be a useful prognostic and predictive marker for CPM-containing chemotherapy in basal-like breast cancer.

## Introduction

Alkylating agents comprise a large class of chemotherapeutic drugs used to treat several types of cancers. Alkylating agents cause cell death by forming cross-links between adjacent strands of DNA via alkylation at the O^6^-position of guanine. O^6^-Methylguanine-DNA methyltransferase (MGMT) is a DNA repair protein that prevents cross-link formation by transferring the alkyl group to an internal cysteine residue, restoring guanine in the DNA and inactivating itself in the process. Various human tumor cell lines and xenografts have been used to demonstrate the association between the activity of MGMT and the ability to withstand methylating agents and alkylating nitrosoureas (1).

Cyclophosphamide (CPM) has been used against a broad spectrum of malignancies. CPM is unique in its lack of alkylating activity, requiring *in vivo* metabolic activation to produce active alkylating compounds (2). In an *in vivo* mouse xenograft study, lung tumor xenografts with high MGMT activity were revealed to be less sensitive to the growth-inhibitory effects of CPM than those with low MGMT activity (3). However, another study of 23 varying xenograft tumors showed no correlation between CPM anti-tumor and MGMT tumor activity (4). In clinical studies, MGMT negativity was associated with significantly increased overall survival (OS) among patients with diffuse large B-cell lymphoma following treatment with CPM and other drugs (5,6). However, in the patients with breast cancer that was treated with neoadjuvant chemotherapy, including CPM, no correlation was observed between MGMT expression and the response to CPM (7). Conversely, a recent study demonstrated the predictive value of MGMT protein expression in breast tumor biopsies obtained prior to CPM-containing neoadjuvant chemotherapy (8).

MGMT is ubiquitously expressed in normal human tissues. Levels of MGMT are known to vary considerably between individuals and organ types, and MGMT activity is usually higher in malignant tissues compared with normal tissues (9). In breast tumors, MGMT expression can be up to 4-fold higher compared with normal breast tissues (10,11). Certain clinical studies have indicated that MGMT expression correlates with the prognosis of breast cancer, whereas other clinical studies have failed to elucidate any association between MGMT expression and OS in patients with breast cancer (7,12–14). The longstanding belief has been that breast tumors are heterogeneous with respect to MGMT expression, however, these contradictory results could be due to the majority of previous studies being evaluated in heterogeneous groups of patients with breast cancer (15).

Gene expression profiling studies on breast tumors have identified 4 main intrinsic molecular subtypes of breast cancer. These are the luminal A, luminal B, human epidermal growth factor receptor-2 (HER2)-enriched and basal-like subtypes (16,17). These subtypes exhibit significant differences in terms of incidence, risk factors, prognosis and response to treatment (18). The prognosis of basal-like cancers tends to be poor. As their response to chemotherapy is poorly understood, optimal chemotherapy regimens and reliable prognostic and predictive factors for these cancers are widely sought after. A recent study indicated that the MGMT promoter methylation status predicts tumor response to alkylating drugs in patients with triple-negative (TN) breast cancer (19). In the present study, immunohistochemical analyses of breast tumor subtypes and MGMT protein expression were conducted using formalin-fixed, paraffin-embedded specimens obtained from patients with breast cancer. The correlation between MGMT expression and prognosis in the subtypes of breast cancer, managed according to common therapeutic protocols, was then investigated. The association between MGMT expression and the clinical outcome was examined to explore the possibility of MGMT expression as a predictive marker in patients with breast cancer treated with CPM-containing chemotherapy.

## Materials and methods

### Patients

Between November 2006 and December 2010, 1,049 patients with breast cancer underwent surgery at Kinki University Hospital (Osaka-Sayama, Osaka, Japan). In total, 635 patients with invasive breast carcinomas, whose resected materials were suitable for further immunohistochemical examinations, were selected (Table I). The ages of the patients ranged between 22 and 89 years (median, 58 years). All patients provided written informed consent for collection of their tissue material and clinical data for research purposes, and the study protocol was approved by the institutional review board (Kinki University Hospital, Osaka-Sayama, Osaka, Japan). None of the patients had distant metastasis or underwent pre-operative therapy. Adjuvant therapy was individually based on indicators of treatment response (hormone and HER2 status) and risk indicators. The patients who underwent breast-conserving surgery received post-operative radiotherapy to the residual breast tissue (50 Gy in 25 fractions).

### Immunohistochemical analysis of breast cancer subtype

Representative portions of formalin-fixed, paraffin-embedded tumor-containing sections were prepared for hematoxylin and eosin staining to confirm invasive carcinoma, and then serial 4-μm thick sections were generated for immunohistochemical analysis. The Allred scoring system was used to measure estrogen receptor (ER) and progesterone receptor (PgR) expression. HER2 analysis was based on the American Society of Clinical Oncology/College of American Pathologists guidelines (20). Immunostaining of cytokeratin (CK) 5/6 and EGFR (HER1) was performed as described previously, and positivity for CK 5/6 and EGFR was evaluated by two investigators who were blinded to the clinical outcomes (21). CK 5/6 was scored as positive when ≥1% of invasive tumor cells exhibited strong cytoplasmic and/or membranous staining. Positivity for EGFR was defined as the detection of ≥10% of cells in the invasive tumors exhibiting strong membrane staining (22).

### Immunohistochemical staining of MGMT

The immunohistochemical procedures were as previously described (23). Briefly, paraffin sections were deparaffinized and antigen retrieval was performed by microwaving the samples. Endogenous peroxidase activities were blocked with 3% hydrogen peroxidase in methanol. Following the blocking of the non-specific binding with 10% normal goat serum, the sections were incubated with a 1:20 dilution of anti-MGMT mouse monoclonal antibody (clone MT3.1; Chemicon, Bioscience Research Reagents, Temecula, CA, USA) for 60 min at room temperature. Staining was achieved using the Envision kit according to the manufacturer’s instructions (Dako, Carpinteria, CA, USA). To assess MGMT immunostaining, each slide was individually reviewed and scored by two investigators. At least 1,000 tumor cell nuclei were evaluated in each specimen, in fields exhibiting the highest density of immunopositive cells. Samples were considered positive when immunoreactivity was detected in >10% of cell nuclei.

### Statistical analysis

Statistical analysis was performed using JMP software, version 8 (SAS Institute, Inc., Cary, NC, USA). Correlations between tumor characteristics and MGMT protein expression were determined using the χ^2^ test. The probabilities of OS and disease-free survival (DFS) were calculated according to the Kaplan-Meier method and were compared using the log-rank test. The OS time was measured from the date of definitive surgery to the date of the last follow-up or mortality from any cause. DFS events included local, regional or distant breast cancer recurrences, second cancer (in the contralateral breast and non-breast cancers) and all mortalities from the time of surgery. All tests were two-sided and P<0.05 was considered to indicate a statistically significant difference.

## Results

### Immunohistochemical subtypes

In total, the 635 cases of invasive carcinoma were classified into 5 clinical subtypes using immunohistochemical markers as follows: Luminal A (ER^+^ and/or PgR^+^ and HER2^−^), luminal B (ER^+^ and/or PgR^+^ and HER2^+^), HER2^+^/ER^−^ (ER^−^, PgR^−^ and HER^+^), basal-like (ER^−^, PgR^−^, HER2^−^, CK 5/6^+^ and/or HER1^+^) and unclassified (negative for all 5 markers) (24). Of the 635 primary tumors analyzed in the present study, 425 (67%) were luminal A, 95 (15%) were luminal B, 47 (7%) were HER2^+^/ER^−^, 48 (7%) were basal-like and 20 (3%) were unclassified. Clinicopathological features were not significantly correlated with immunohistochemical subtypes.

### Immunohistochemical analysis of MGMT protein expression

**Figure 1:**
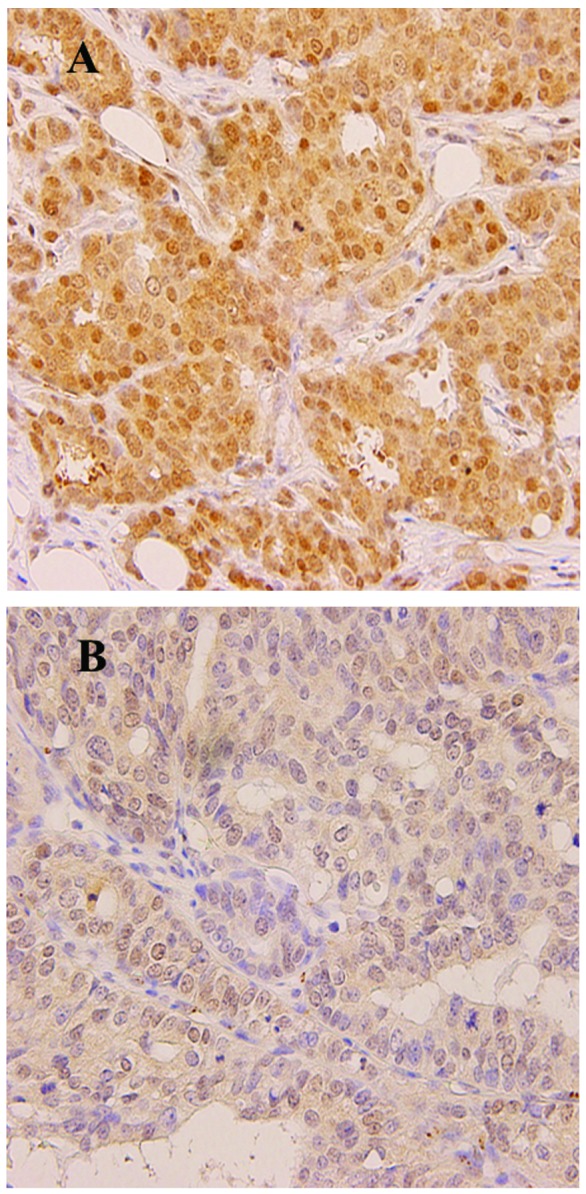
Representative photomicrographs of immunohistochemical staining for MGMT in breast cancer (magnification, ×100). Positive staining is denoted by brown-stained nuclei and extremely weak cytoplasmic staining. (A) MGMT positivity (fraction of positive cells >10%). (B) MGMT negativity. MGMT, O^6^-methylguanine-DNA methyltransferase.

Immunohistochemistry revealed intense nuclear staining and weak cytoplasmic staining for MGMT in the tumor cells (Fig. 1). Tumors were defined as MGMT-positive when >10% of cells exhibited nuclear staining. MGMT positivity was identified in 398 (63%) of 635 patients with breast cancer. The characteristics of the 635 patients according to the MGMT immunoreactivity are presented in Table II. The frequency of MGMT protein expression was significantly higher in the tumors with no lymph node metastasis compared with the tumors positive for lymph node metastasis. The MGMT status was significantly correlated with the hormone receptor status. A significant inverse correlation between the MGMT status and the HER2 and HER1 status was observed (Table II). MGMT positivity was identified in 68% of luminal A and 67% of luminal B tumors, whereas MGMT negativity was significantly more common in HER2^+^/ER^−^ tumors. In total, 22 (46%) basal-like tumors were MGMT-positive. MGMT expression was not significantly correlated with breast cancer subtype.

### Clinical significance of MGMT protein expression

The patients in the study received systemic therapies based on indicators of treatment response (tumor hormone and HER2 status of the tumor) and risk. Following surgery, 2% of patients received no adjuvant therapy, 60% received endocrine therapy, 17% received chemotherapy and 21% received endocrine therapy and chemotherapy. The median follow-up time after surgery was 4.6 years (range, 0.9–7.8 years). OS and DFS rates differed significantly according to the breast cancer subtype, and were significantly worse for patients with basal-like tumors than for those with luminal A tumors.

**Figure 2:**
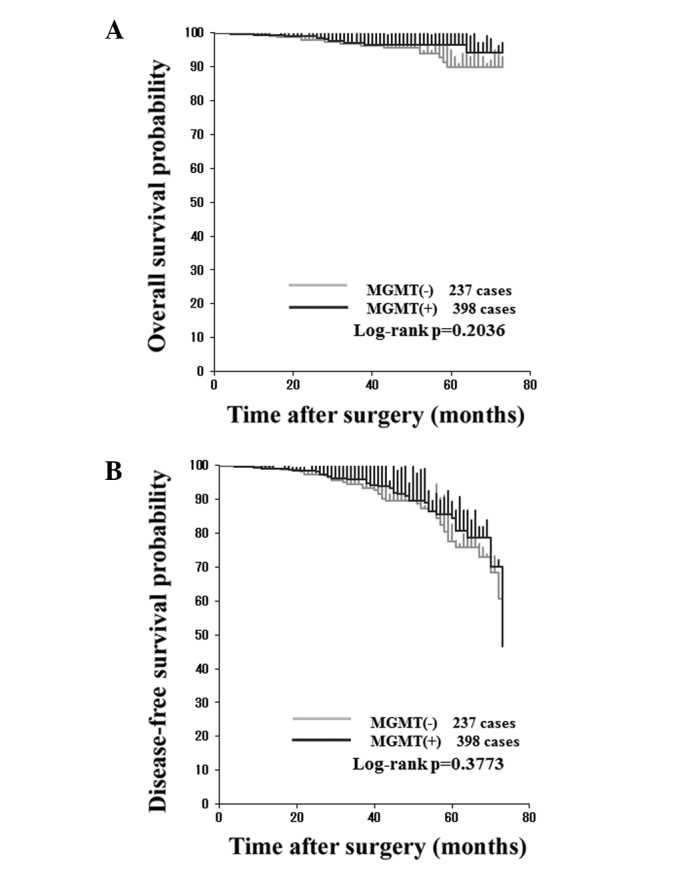
Kaplan-Meier (A) overall survival (OS) and (B) disease-free survival (DFS) curves of patients with breast cancer according to MGMT status. MGMT, O^6^-methylguanine-DNA methyltransferase.

**Figure 3:**
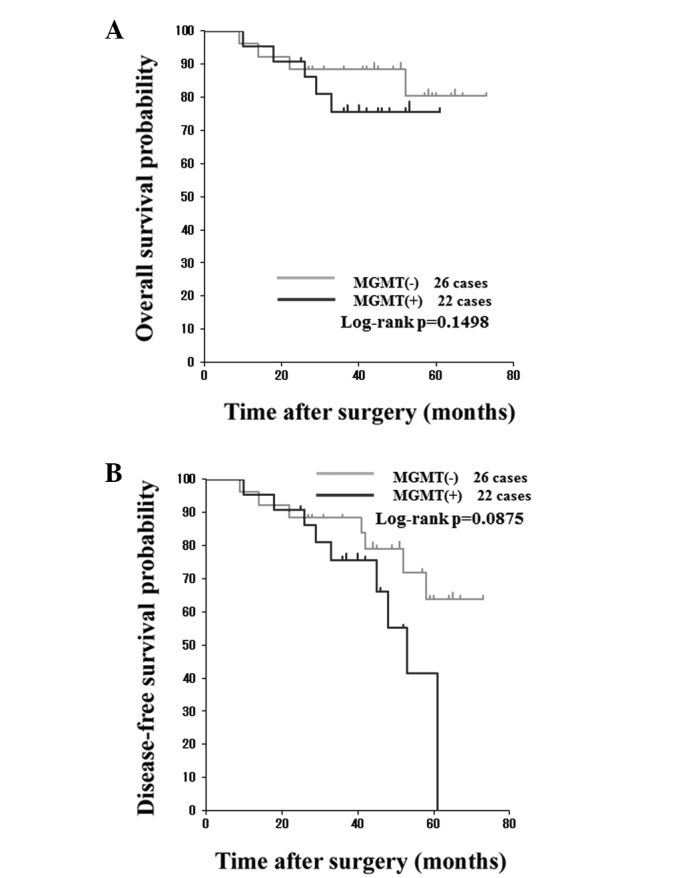
Kaplan-Meier (A) overall survival (OS) and (B) disease-free survival (DFS) curves of patients with basal-like cancers according to MGMT status. MGMT, O^6^-methylguanine-DNA methyltransferase.

The prognostic significance of MGMT protein expression was analyzed. OS and DFS rates did not significantly differ between the patients with MGMT-positive tumors and those with MGMT-negative tumors (Fig. 2). In comparing the clinical outcomes of the patients with MGMT-positive tumors with those of the patients with MGMT-negative tumors according to immunohistochemical subtypes, no statistically significant difference in OS and DFS rates was found according to MGMT expression for luminal A, luminal B or HER2^+^/ER^−^ tumors. However, Kaplan-Meier estimates revealed that the patients with MGMT-negative basal-like tumors tended to have better DFS, but not OS, compared with those with MGMT-positive tumors (Fig. 3).

**Figure 4:**
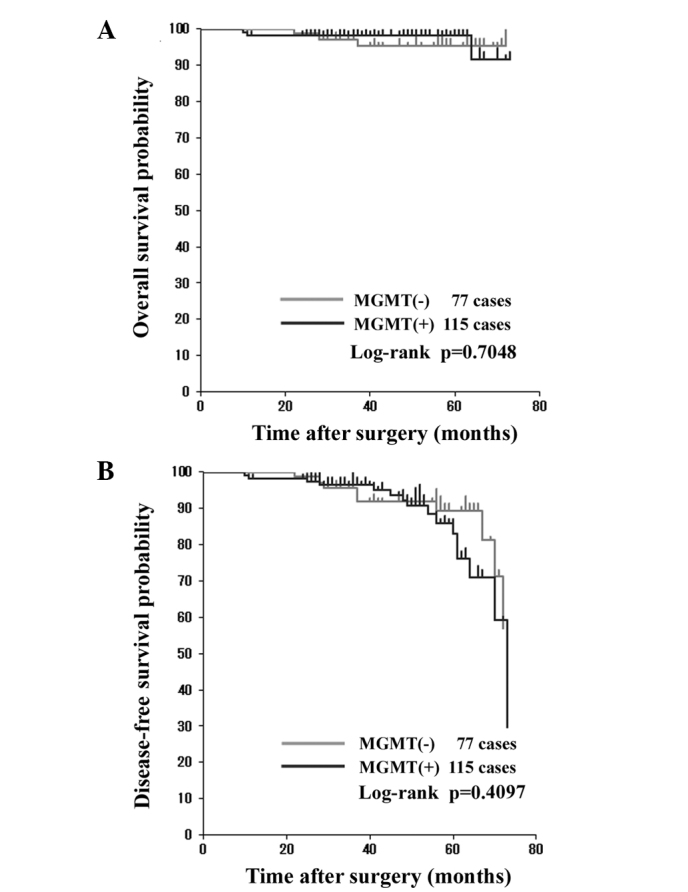
Kaplan-Meier (A) overall survival (OS) and (B) disease-free survival (DFS) curves of patients with breast cancer treated with CPM-containing chemotherapy according to MGMT status. CPM, cyclophosphamide; MGMT, O^6^-methylguanine-DNA methyltransferase.

**Figure 5:**
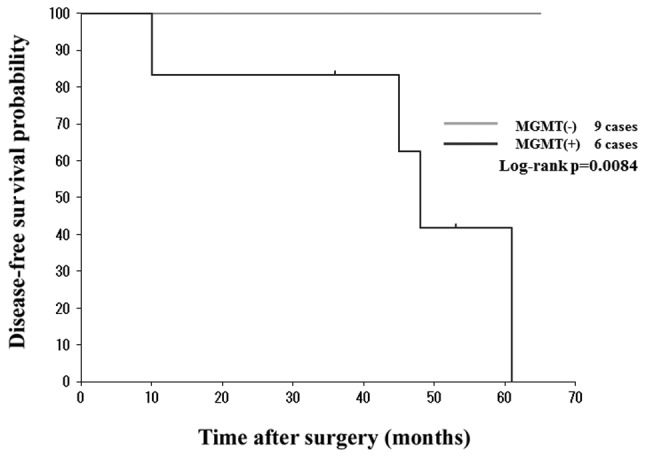
Kaplan-Meier disease-free survival (DFS) curves of patients with basal-like cancers treated with CPM-containing chemotherapy according to MGMT status. CPM, cyclophosphamide; MGMT, O^6^-methylguanine-DNA methyltransferase.

**Figure 6:**
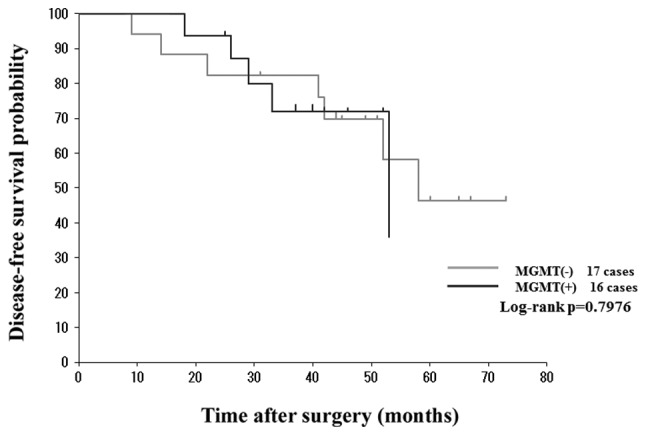
Kaplan-Meier disease-free survival (DFS) curves of patients with basal-like cancers who did not receive CPM-containing chemotherapy according to MGMT status. CPM, cyclophosphamide; MGMT, O^6^-methylguanine-DNA methyltransferase.

**Figure 7:**
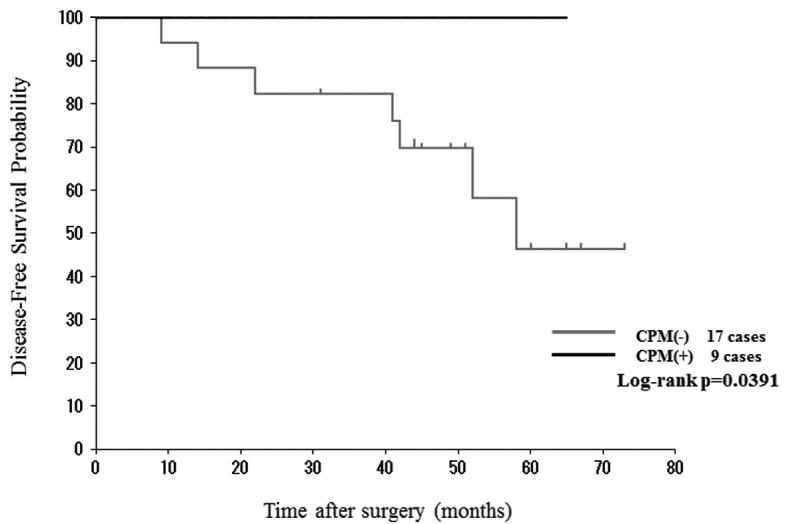
Kaplan-Meier disease-free survival (DFS) curves of patients with MGMT-negative basal-like cancers treated with and without CPM-containing chemotherapy. CPM, cyclophosphamide; MGMT, O^6^-methylguanine-DNA methyltransferase.

The potential of MGMT protein expression as a predictive marker for the efficacy of alkylating agents was investigated. CPM-containing chemotherapy was administered to 112 (26%), 38 (40%), 22 (47%) 15 (31%) and 5 (25%) patients with luminal A, luminal B, HER2^+^/ER^−^, basal-like and unclassified tumors, respectively. Of the 192 patients who received CPM-containing chemotherapy, 115 exhibited MGMT-positive tumors. Kaplan-Meier estimates of OS and DFS rates for the patients who received CPM revealed no significant differences according to MGMT expression (Fig. 4). The clinical outcomes of patients with luminal A, luminal B or HER2^+^/ER^−^ tumors treated with CPM-containing regimens were analyzed. OS and DFS rates in the patients with luminal A, luminal B or HER2^+^/ER^−^ tumors did not differ significantly according to the MGMT status. Among the basal-like tumors from the 15 patients who received CPM-containing regimens as adjuvant chemotherapy, 6 were MGMT-positive and 9 were MGMT-negative. When analyzing the clinical outcomes of these patients, no recurrence was observed in those with MGMT-negative tumors, however, 4 of the 6 patients with MGMT-positive tumors had breast cancer recurrences. Patients with MGMT-negative tumors had a significantly improved DFS rate compared with the patients with MGMT-positive tumors (Fig. 5). By contrast, Kaplan-Meier estimates of DFS in the 33 patients with basal-like tumors who were not administered CPM as an adjuvant therapy revealed no statistical differences according to MGMT expression (Fig. 6). Of the 48 patients with basal-like tumors, 26 had MGMT-negative tumors. Among these 26 patients, 9 received CPM-containing adjuvant chemotherapy. Compared with the patients treated with CPM-containing chemotherapy, the patients who did not receive CPM-containing chemotherapy had a significantly poor prognosis (Fig. 7).

## Discussion

Numerous methods have been used to analyze MGMT status in a wide-spectrum of human tumors. In the present study, MGMT status was assessed in patients with breast cancer by analyzing MGMT protein expression using an immunohistochemical method, which can be easily performed without specific equipment, even on archived formalin-fixed, paraffin-embedded specimens, together with other immunohistochemical studies for tumor diagnosis. MGMT protein expression was studied in a large series of cases from a single institution using an immunohistochemical method. In total, 63% of the 635 patients with breast cancer harbored MGMT-positive tumors. To the best of our knowledge, using various anti-MGMT antibodies and applying diverse criteria for MGMT immunopositivity, immunohistochemical studies concerning MGMT protein expression in breast cancer have demonstrated that 54–81% of breast cancers are MGMT-positive (8,12–14,25,26).

To clarify the clinical relevance of MGMT expression in patients with breast cancer, the association between clinicopathological factors and MGMT status in breast cancers was investigated first. Breast cancers with no lymph node metastasis were more likely to be MGMT-positive than those with positive lymph nodes. MGMT status showed a strong correlation with ER and PgR status and a significant, inverse correlation with HER2 status. The patients with ER-positive disease received endocrine therapy, and anti-HER2 therapy was indicated in the patients with HER2-positive disease. However, MGMT expression was not correlated with OS or DFS among the patients with breast cancer in the present study.

Next, MGMT protein expression as a possible prognostic factor in breast cancer subtypes was examined using the Kaplan-Meier method. Although Neto *et al* did not analyze clinical outcome of patients with breast cancer, the study indicated that MGMT expression was correlated with factors for a poor prognosis, including the molecular phenotype of breast cancers (14). The present study found no significant association between MGMT status and the clinical outcome of patients with luminal A, luminal B, HER2^+^/ER^−^ and basal-like subtypes. It was therefore concluded that MGMT protein expression generally has no prognostic value for breast cancers.

In the present study, basal-like cancer was defined as ER^−^, PgR^−^, HER2^−^, CK 5/6^+^ and/or EGFR^+^ tumors using immunohistochemical surrogate markers (24). Patients with basal-like breast cancers do not benefit from molecular-targeted treatments, including endocrine therapy or trastuzumab. A previous *in vivo* study demonstrated that chemosensitivity in human cells lacking BRCA1, and to a certain extent other TN cells, may be sensitive to drugs that cause double-strand breaks in DNA, including alkylating agents (27). Although basal-like breast cancer is not identical to TN tumors, previous retrospective studies have indicated that classical CPM, methotrexate and fluorouracil chemotherapy may be beneficial for breast cancer patients with TN phenotypes (28–30). In the present study, approximately one-third of the patients with basal-like breast cancer received CPM-containing adjuvant chemotherapy. Among the patients with basal-like breast cancer treated with a CPM-containing regimen, those with MGMT-negative tumors had a better DFS rate compared with those with MGMT-positive tumors. Furthermore, among the patients with basal-like breast cancer with an MGMT-negative phenotype, those who received CPM-containing chemotherapy exhibited an improved DFS rate compared with those who received chemotherapy without CPM. However, the clinical outcomes of patients with other subtypes of breast cancer were independent of the MGMT status, irrespective of the treatment with CPM-containing chemotherapy. Taken together, the results of the present study indicate that MGMT protein expression could be a useful prognostic and predictive marker of patients with basal-like breast cancer who are treated with CPM-containing chemotherapy.

The majority of chemotherapeutic regimens that have been used to treat all stages of breast cancer have used CPM as an integral component. In the liver, CPM undergoes metabolic activation to form phosphoramide mustard and acrolein (2). Phosphoramide mustard is believed to produce interstrand cross-links between the N7-position of guanines on opposite DNA strands, which are considered to be the major cytotoxic lesions. Previous studies have indicated that acrolein, and not phosphoramide mustard, is the mutagenic/toxic metabolite of CPM, with MGMT being involved in its repair (31). The association between MGMT expression and the resistance to CPM has been debated (32). It is clear that drug resistance in cancer is a multifactorial process. High intracellular aldehyde dehydrogenase activity is reported to be an important determinant of CPM sensitivity (33). The decreased expression or mutation of p53 has also been associated with chemoresistance (34). Other postulated mechanisms of resistance include the increased expression of the glutathione S-transferase group of enzymes, resulting in an increased deactivation of potentially damaging metabolites of CPM and a depletion of cellular glutathione (32). These various mechanisms possibly operate simultaneously, and the precise contribution of each is thus difficult to define.

Finally, the present study demonstrated that the MGMT expression level and improvement in clinical outcomes are significantly correlated among patients with basal-like breast cancers who receive CPM-containing adjuvant chemotherapy. At present, the exact reasons why MGMT protein expression affects CPM-containing chemotherapy in these patients are not known. As Lehmann *et al* (35) reported two different subtypes of basal-like breast cancer using gene expression analysis, our future studies will focus on elucidating the role of MGMT in the anti-tumor effect and toxicity of CPM, and identifying a subset of basal-like breast cancer that exhibits sensitivity to CPM.

## Figures and Tables

**Table I:** Patient characteristics.

	Total patients (n=635)	
	
Variables	No.	%
Menopausal status		
Pre	202	32
Post	433	68
TNM stage		
I	254	40
II	326	51
III	55	9
Node status		
Negative	400	63
Positive	235	37
Estrogen receptor		
Negative	122	19
Positive	513	81
Progesterone receptor		
Negative	162	26
Positive	473	74
HER2		
Negative	493	78
Positive	142	22

TNM, tumor-node-metastasis; HER2, human epidermal growth factor receptor-2.

**Table II:** Association between MGMT status and clinicopathological variables.

	MGMT, n (%)		
		
Variables	Positive	Negative	P-value
TNM stage			
I	172 (68)	82 (32)	0.09
II	195 (60)	131 (40)	
III	31 (56)	24 (44)	
Node status			
Negative	264 (66)	136 (34)	0.02
Positive	134 (57)	101 (43)	
Estrogen receptor			
Negative	49 (40)	73 (60)	0.001
Positive	349 (68)	164 (32)	
Progesterone receptor			
Negative	76 (47)	86 (53)	0.001
Positive	322 (68)	151 (32)	
HER2			
Negative	320 (65)	173 (35)	0.03
Positive	78 (55)	64 (45)	
CK 5/6			
Negative	372 (63)	222 (37)	0.87
Positive	27 (66)	14 (34)	
HER1			
Negative	371 (65)	203 (35)	0.01
Positive	28 (46)	33 (54)	
Subtype			
Luminal A	291 (68)	134 (32)	0.001
Luminal B	64 (67)	31 (33)	
HER2^+^/ER^−^	14 (30)	33 (70)	
Basal-like	22 (46)	26 (54)	
Unclassified	7 (35)	13 (65)	

MGMT, O^6^-methylguanine-DNA methyltransferase; TNM, tumor-node-metastasis; HER1, human epidermal growth factor receptor-1; ER, estrogen receptor; CK, cytokeratin.
